# Contrast MR-Based Radiomics and Machine Learning Analysis to Assess Clinical Outcomes following Liver Resection in Colorectal Liver Metastases: A Preliminary Study

**DOI:** 10.3390/cancers14051110

**Published:** 2022-02-22

**Authors:** Vincenza Granata, Roberta Fusco, Federica De Muzio, Carmen Cutolo, Sergio Venanzio Setola, Federica dell’ Aversana, Alessandro Ottaiano, Antonio Avallone, Guglielmo Nasti, Francesca Grassi, Vincenzo Pilone, Vittorio Miele, Luca Brunese, Francesco Izzo, Antonella Petrillo

**Affiliations:** 1 Division of Radiology, Istituto Nazionale Tumori IRCCS Fondazione Pascale–IRCCS di Napoli, 80131 Naples, Italy; s.setola@istitutotumori.na.it (S.V.S.); a.petrillo@istitutotumori.na.it (A.P.); 2 Medical Oncology Division, Igea SpA, 80013 Napoli, Italy; r.fusco@igeamedical.com; 3 Department of Medicine and Health Sciences V. Tiberio, University of Molise, 86100 Campobasso, Italy; demuziofederica@gmail.com (F.D.M.); luca.brunese@unimol.it (L.B.); 4 Department of Medicine, Surgery and Dentistry, University of Salerno, 84084 Salerno, Italy; carmencutolo@hotmail.it (C.C.); v.pilone@istitutotumori.na.it (V.P.); 5 Division of Radiology, Università Degli Studi Della Campania Luigi Vanvitelli, 80138 Naples, Italy; federica.dellaversana@unicampania.it (F.d.A.); francesca.grassi1@studenti.unicampania.it (F.G.); 6 Division of Abdominal Oncology, Istituto Nazionale Tumori IRCCS Fondazione Pascale–IRCCS di Napoli, 80131 Naples, Italy; a.ottaiano@istitutotumori.na.it (A.O.); a.avallone@istitutotumori.na.it (A.A.); g.nasti@istitutotumori.na.it (G.N.); 7 Division of Radiology, Azienda Ospedaliera Universitaria Careggi, 50134 Florence, Italy; mielev@aou-careggi.toscana.it; 8 Italian Society of Medical and Interventional Radiology (SIRM), SIRM Foundation, Via della Signora 2, 20122 Milan, Italy; 9 Division of Epatobiliary Surgical Oncology, Istituto Nazionale Tumori IRCCS Fondazione Pascale–IRCCS di Napoli, 80131 Naples, Italy; f.izzo@istitutotumori.na.it

**Keywords:** colorectal liver metastasis, magnetic resonance imaging, radiomics, pattern recognition, outcome prediction

## Abstract

**Simple Summary:**

The objective of the study was to evaluate the radiomics features obtained by contrast MRI studies as prognostic biomarkers in colorectal liver metastases patients to predict clinical outcomes following liver resection. We demonstrated a good performance considering the single textural significant metric in the identification of front of tumor growth (expansive versus infiltrative) and tumor budding (high grade versus low grade or absent), in the recognition of mucinous type and in the detection of recurrences. Moreover, considering linear regression models or neural network classifiers in a multivariate approach was possible to increase the performance in terms of accuracy, sensitivity, and specificity.

**Abstract:**

Purpose: To assess radiomics features efficacy obtained by arterial and portal MRI phase in the prediction of clinical outcomes in the colorectal liver metastases patients, evaluating recurrence, mutational status, pathological characteristic (mucinous and tumor budding) and surgical resection margin. Methods: This retrospective analysis was approved by the local Ethical Committee board, and radiological databases were used to select patients with colorectal liver metastases with pathological proof and MRI study in a pre-surgical setting after neoadjuvant chemotherapy. The cohort of patients included a training set (51 patients with 61 years of median age and 121 liver metastases) and an external validation set (30 patients with single lesion with 60 years of median age). For each segmented volume of interest on MRI by two expert radiologists, 851 radiomics features were extracted as median values using the PyRadiomics tool. Non-parametric Kruskal-Wallis test, intraclass correlation, receiver operating characteristic (ROC) analysis, linear regression modelling and pattern recognition methods (support vector machine (SVM), k-nearest neighbors (KNN), artificial neural network (NNET), and decision tree (DT)) were considered. Results: The best predictor to discriminate expansive versus infiltrative tumor growth front was wavelet_LHH_glrlm_ShortRunLowGrayLevelEmphasis extracted on portal phase with accuracy of 82%, sensitivity of 84%, and specificity of 77%. The best predictor to discriminate tumor budding was wavelet_LLH_firstorder_10Percentile extracted on portal phase with accuracy of 92%, a sensitivity of 96%, and a specificity of 81%. The best predictor to differentiate the mucinous type of tumor was the wavelet_LLL_glcm_ClusterTendency extracted on portal phase with accuracy of 88%, a sensitivity of 38%, and a specificity of 100%. The best predictor to identify the recurrence was the wavelet_HLH_ngtdm_Complexity extracted on arterial phase with accuracy of 90%, a sensitivity of 71%, and a specificity of 95%. The best linear regression model was obtained in the identification of mucinous type considering the 13 textural significant metrics extracted by arterial phase (accuracy of 94%, sensitivity of 77% and a specificity of 99%). The best results were obtained in the identification of tumor budding with the eleven textural significant features extracted by arterial phase using a KNN (accuracy of 95%, sensitivity of 84%, and a specificity of 99%). Conclusions: Our results confirmed the capacity of radiomics to identify as biomarkers and several prognostic features that could affect the treatment choice in patients with liver metastases in order to obtain a more personalized approach.

## 1. Introduction

Radiomics is a promising area that investigates the capability of quantitative features extracted by medical images as biomarkers to assess the biology of pathological processes at microscopic levels. These data can be converted into image-based marks to spread diagnostic, prognostic and predictive accuracy in oncological setting [1,2,3,4,5,6,7,8]. Radiomics could theoretically support tumor detection, evaluation of prognosis, estimate treatment response [9,10,11,12,13,14]. Radiomics is designed to be used in decision support of precision medicine, using standard of care images that are routinely acquired in clinical practice. It presents a cost-effective and highly feasible addition for clinical decision support. Moreover, this analysis non-invasively characterize the overall tumor accounting for heterogeneity, interrogating the entire tumor allows the expression of microscopic genomic and proteomics patterns in terms of macroscopic image-based features [15,16,17,18]. Moreover, this analysis gives prognostic and/or predictive biomarker allowing for a fast, low-cost, and repeatable tool for longitudinal monitoring [19,20].

The association of radiomics and molecular features, so named radiogenomics, shows clear effects for management of cancer patients. Although several studies have assessed the rule of radiogenomics in hepatocellular carcinoma, only a few have tested the radiomics rule in colorectal cancer metastatic lesions in the liver [1,2,3]. Today, radiologists play an important role in the multidisciplinary team of colorectal patients with liver metastases. During the staging and surveillance phase, it is critical to identify all liver lesions, since this is related to proper patient management. Additionally, after conversion therapy, all lesions assessed at first exam should be re-evaluated to identify responders and non-responders as soon as possible [21,22,23,24,25]. Although computed tomography (CT) is habitually the diagnostic tool employed for staging and surveillance, magnetic resonance imaging (MRI) is a valuable diagnostic technique in oncologic settings, since it is the only technique that allows evaluating of morphological and functional features of tumor status, providing quantitative parameters that improve the characterization of a lesion and the assessment after therapy [21,22,23,24]. Moreover, several liver-specific contrast agents have been inserted to improve the hepatic lesions detection and characterization. Gadobenate dimeglumine (Gd-BOPTA) and gadolinium ethoxybenzyl diethylenetria-mine pentaacetic acid (Gd-EOB-DTPA) allow obtaining information about the vascularization of lesions in the different phases of contrast circulation and functional parameters in the delayed hepatobiliary phase (EOB-phase).

In this scenario, the possibility to compare radiomic data extracted by MRI in the identification of recurrence, mutational status, pathological characteristics (mucinous and tumor budding), and surgical resection margin could provide significant benefits respect to qualitative evaluation. In fact, radiomics predictors could permit an effective treatment selection in the perspective of personalized medicine, treatment response prediction, in the differentiation of favorable subsets of patients from those with poor prognosis, and selecting patients that may benefit from surgical treatment. In the present study, we assessed the radiomics features efficacy obtained by contrast (arterial and portal phase) MRI to predict clinical outcomes following liver resection in colorectal liver metastases patients.

## 2. Materials and Methods

### 2.1. Dataset Characteristics

This study was approved by the local ethical committee board that renounces the patient informed consent due to the retrospective nature of the study. The study was performed in accordance with relevant guidelines and regulations.

Patient selection was made considering internal radiological databases in the period from January 2018 to May 2021 using the following criteria: (1) liver pathological proven metastases; (2) contrast MRI study in pre-surgical setting after neoadjuvant chemotherapy; (3) MR images of high quality and (4) a follow-up CT scan of at least six months after surgery. The exclusion criteria were: (1) discordance among the imaging diagnosis and the pathological ones, (2) no contrast MRI studies and (c) no high-quality MR images.

The analyzed patients included a training set and an external validation set. The internal training set included 51 patients (18 women and 33 men) with 61 years of median age (range 35–82 years) and 121 liver metastases. The validation cohort, provided by “Careggi Hospital”, Florence, Italy, consisted of a total of 30 patients with single lesion (10 women and 20 men) with 60 years of median age (range 40–78 years). The patient characteristics are summarized in Table 1.

### 2.2. MR Imaging Protocol

A Magnetom Symphony 1.5 T scanner (Siemens, Erlangen, Germany) and a Magnetom Aera (Siemens) 1.5 T scanner equipped with an 8-element body and phased array coils were used to acquire an MRI study that includes sequences before and after intravenous (IV) contrast agent (CA) injection.

In this study, radiomic features extraction was made on volumetric interpolated breath-hold examination (VIBE) T1-weighted SPAIR with controlled respiration used to acquire images after IV CA injection with a liver-specific CA (0.1 mL/kg of Gd-EOB-BPTA, Primovist, Bayer Schering Pharma, Berlin, Germany) as descripted in [26,27].

The VIBE T1-W sequence was acquired with two different flip angles (10 and 30 degrees). A power injector (Spectris Solaris^®^ EP MR, MEDRAD, Inc., Indianola, IA, USA) was used to administer the CA at an infusion rate of 2 mL/s. VIBE T1-w images were acquired in four different phases: arterial phase (35 s delay), portal venous phase (90 s), late/transitional phase (120 s), and hepatobiliary excretion phase (20 min). MRI protocol details are reported in Table 2.

### 2.3. Follow-Up CT Scan

CT studies were performed using a scanner with 64 detectors (Optima 660, GE Healthcare, Chicago, IL, USA). The scan data was 120 kVp, 100–470 mA (NI 16.36), slice thickness was 2.5 mm, and table speed/rotation was 0.984/1 mm. The liver protocol included a quadruple phase protocol, counting unenhanced, arterial, portal, and equilibrium phases. A non-ionic contrast agent (120 mL of iomeprol, Iomeron 400, Bracco, Milan, Italy) was injected at a rate of 3 mL/s using an automatic power injector (Empower CTA, EZ-EM Inc., New York, NY, USA). The arterial phase was started 19 s after the descending aorta attenuation reached 100 HU, measured by the bolus localization method.

### 2.4. Image Processing

Regions of interest (ROIs) were manually drawn slice-by-slice by two expert radiologists with 22 and 15 years of abdominal imaging experience, respectively, first separately and then together and in accordance with each other. Region of interest segmentation was performed avoiding encircling any distortion artefacts. For each volume of interest, radiomics features were extracted as median values, reducing the possible influence by artefacts.

The segmentation was performed on arterial phase and portal phase of VIBE T1-W_FA10. For these reasons, we obtained the results both on arterial phase volume and on portal phase volume.

**Figure 1:**
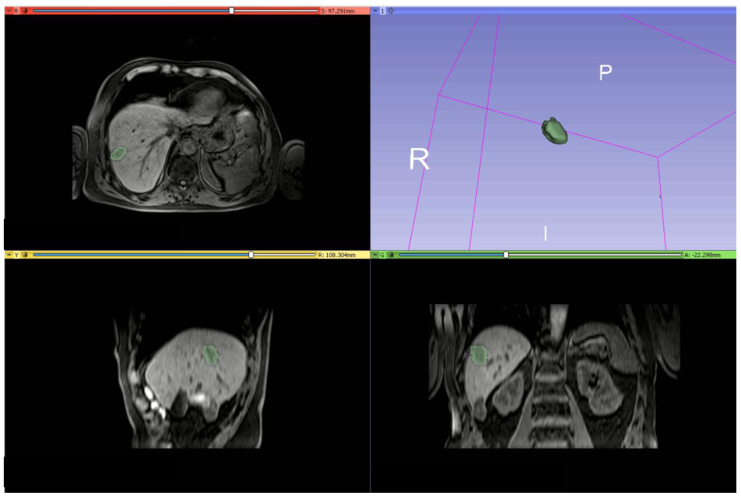
An example of manual definition of the ROIs made using the segmentation tool of 3DSlicer on VIBE T1-W_FA10.

Manual definition of the ROIs was made using the segmentation tool of 3DSlicer 4.11 (Figure 1) [https://www.slicer.org/, accessed on 20 December 2021].

### 2.5. MRI Post-Processing with Pyradiomic Tool

Eight hundred fifty-one radiomic features were extracted using PyRadiomics v3.0.1 [28] and included first-order statistics, shape-based (3D) metrics, shape-based (2D) features, gray level co-occurence matrix features, gray level run length matrix features, gray level size zone matrix features, neighboring gray tone difference matrix features and gray level dependence matrix parameters. The extracted features are in compliance with feature definitions as described by the Imaging Biomarker Standardization Initiative (IBSI) [29] and reported in [https://readthedocs.org/projects/pyradiomics/downloads/, accessed on 20 December 2021]. Radiomics analysis was performed blinded to the clinical and pathological data.

### 2.6. Statistical Analysis

Statistical analysis included univariate and multivariate approaches.

#### 2.6.1. Univariate Analysis

The observer variability assessment was performed by calculating the intraclass correlation coefficient.

A non-parametric Kruskal-Wallis test was performed to identify statistically significant differences among clinical parameters and radiomic metrics of two groups (front of tumor growth: expansive versus infiltrative; tumor budding: high-grade versus low-grade or absent; mucinous type; and presence of recurrence).

Receiver operating characteristic (ROC) analysis was performed, and the Youden index was used to individuate the optimal cutoff value for each feature in order to calculate area under the ROC curve (AUC), sensitivity, specificity, positive predictive value (PPV), negative predictive value (NPV) and accuracy.

The McNemar test was used to calculate statistically significant differences among dichotomy data of the performance results.

#### 2.6.2. Multivariate Analysis

A multivariate analysis was performed in order to identify the combinations of variables which best predict the outcomes: (1) front of tumor growth: expansive versus infiltrative; (2) tumor budding: high-grade versus low-grade or absent; (3) mucinous type; and (4) presence of recurrence.

Given the high number of textural features, a first selection of variables was made based on the results obtained from the univariate analysis (Table 3). Therefore, there was no waiting for overfitting in our study because adequate feature selection was made according to sample size.

Linear regression modelling was used to assess the best linear combination of features considered as predictors for each outcome (Table 3). The linear regression model was used to assess the accuracy of linear combination, and ROC analysis with Youden index was used to identify the optimal cut-off value, sensitivity, specificity, PPV, and NPV.

Moreover, pattern recognition methods were used in the context of a multivariate artificial intelligence approach. The tested classifiers with a 10-k fold cross-validation were support vector machine (SVM), k-nearest neighbors (KNN), artificial neural network (NNET), and decision tree (DT)). A description of classifiers can be found in [30]. The best classifier was chosen considering the highest area under ROC curve and highest accuracy. An external validation cohort was used to validate the findings of the best classifier found in the training step.

The statistical analyses were performed using the Statistics Toolbox and Machine Toolbox of MATLAB R2021b (MathWorks, Natick, MA, USA) considering a *p* value ≤ 0.05 as significant.

## 3. Results

### 3.1. Univariate Analysis Findings

The intraclass correlation coefficients median value for extracted features was 0.94 (range 0.88–0.98). The size of the lesion did not affect the values of the extracted metrics (*p*-value > 0.05 at the Kruskal-Wallis test considering lesions < 2 cm and ≥ 2 cm).

Among significant features to differentiate the tumor growth front in the arterial phase, 7 textural parameters obtained an accuracy ≥ 75% Among these 7 features, the best performance to discriminate expansive versus infiltrative front of tumor growth was obtained by the wavelet_LHH_glrlm_ShortRunLowGrayLevelEmphasis with accuracy of 79%, sensitivity of 95%, specificity of 51%, PPV and NPV of 77% and 85%, respectively, and a cut-off value of 0.12 (Table 4).

Among significant features to differentiate the front of tumor growth in portal phase, 9 textural parameters obtained an accuracy ≥ 80%. Among these 9 features, the best performance to discriminate expansive versus infiltrative front of tumor growth was obtained by the wavelet_LHH_glrlm_ShortRunLowGrayLevelEmphasis (the same feature of previous case) with accuracy of 82%, sensitivity of 84%, specificity of 77%, PPV and NPV of 85% and 74%, respectively, and a cut-off value of 0.12 (Table 4).

Among significant features to differentiate the tumor budding on arterial phase, 11 textural parameters obtained an accuracy ≥ 80%. Among these 11 features, the best performance to discriminate tumor budding was obtained by the wavelet_LHH_firstorder_Minimum with accuracy of 86%, sensitivity of 98%, specificity of 52%, PPV and NPV of 85% and 89%, respectively, and a cut-off value of −41.76 (Table 4).

Among significant features to differentiate the tumor budding in the portal phase, 13 textural parameters obtained an accuracy ≥ 85%. Among these 13 features, the best performance to discriminate tumor budding was obtained by the wavelet_LLH_firstorder_10Percentile with accuracy of 92%, sensitivity of 96%, specificity of 81%, PPV and NPV of 93% and 86%, respectively, and a cut-off value of −37.14 (Table 4).

Among significant features to differentiate the mucinous type of tumor in the arterial phase, 13 textural parameters obtained an accuracy ≥ 80%. Among these 13 features, the best performance to differentiate the mucinous type of tumor was obtained by the wavelet_HLH_glszm_LargeAreaHighGrayLevelEmphasis with accuracy of 85%, sensitivity of 35%, specificity of 99%, PPV and NPV of 90% and 85%, respectively, and a cut-off value of −0.02 (Table 4).

Among significant features to differentiate the mucinous type of tumor in the portal phase, 12 textural parameters obtained an accuracy ≥ 85%. Among these 12 features, the best performance to differentiate the mucinous type of tumor was obtained by the wavelet_LLL_glcm_ClusterTendency with accuracy of 88%, sensitivity of 38%, specificity of 100%, PPV and NPV of 100% and 86%, respectively, and a cut-off value of 408.22 (Table 4).

Among significant features to identify tumor recurrence in the arterial phase, 10 textural parameters obtained an accuracy ≥ 80%. Among these 10 features, the best performance to identify tumor recurrence was obtained by the wavelet_HLH_ngtdm_Complexity with accuracy of 90%, sensitivity of 71%, specificity of 95%, PPV and NPV of 79% and 90%, respectively, and a cut-off value of 3.34 (Table 4).

Among significant features to identify tumor recurrence in the portal phase, 11 textural parameters obtained an accuracy ≥ 85%. Among these 11 features, the best performance to identify tumor recurrence was obtained by the LHL_glcm_Correlation with accuracy of 89%, sensitivity of 71%, specificity of 94%, PPV and NPV of 81% and 90%, respectively, and a cut-off value of 1.54 (Table 4).

### 3.2. Multivariate Analysis Findings

#### 3.2.1. Linear Regression Analysis Findings

**Figure 2:**
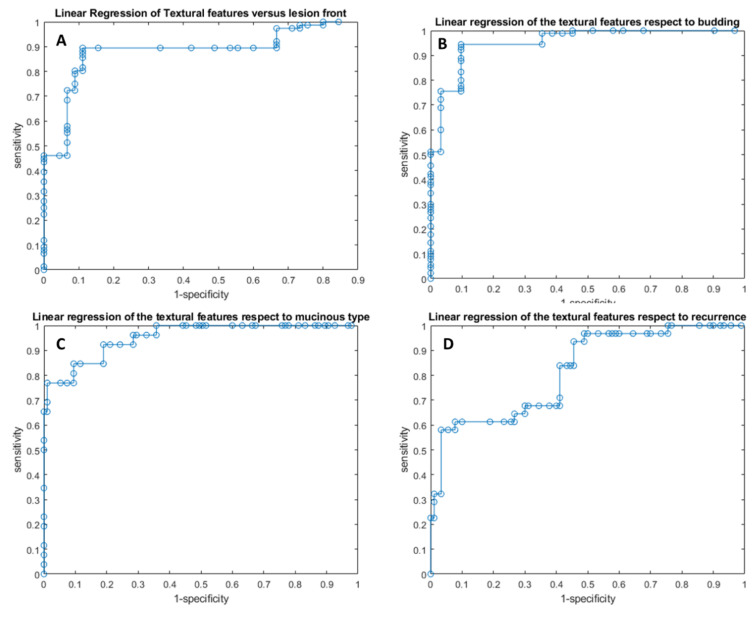
ROC curves of linear regression analysis with respect to the front of tumor growth (**A**), tumor budding (**B**), tumor mucinous type (**C**), and the recurrence presence (**D**) obtained considering significant features extracted by arterial phase.

**Figure 3:**
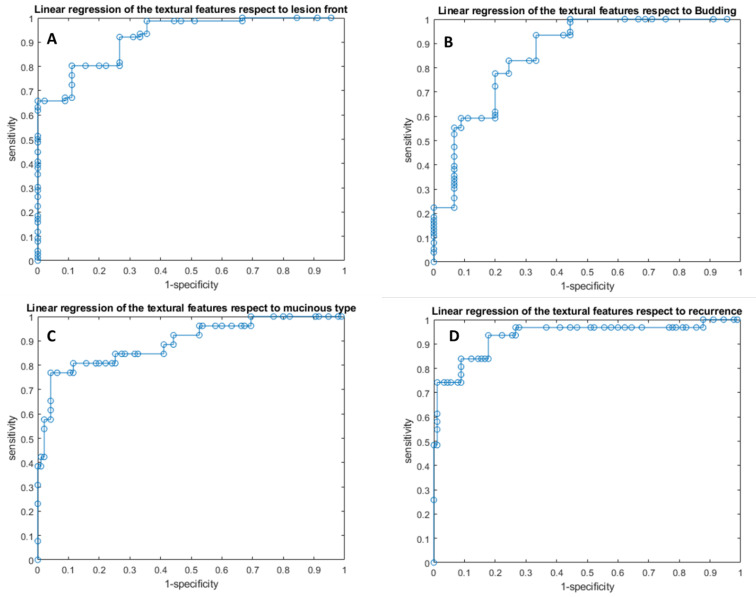
ROC curves of linear regression analysis with respect to the front of tumor growth (**A**), tumor budding (**B**), tumor mucinous type (**C**), and the recurrence presence (**D**) obtained considering significant features extracted by portal phase.

Linear regression models obtained good results in each considered classification problem (1. Front of tumor growth: expansive versus infiltrative; 2. tumor budding: high-grade versus low-grade or absent; 3. mucinous type and 4. presence of recurrence) with accuracy in the range of 83−94% (Table 5 and Table 6, Figure 2 and Figure 3). The best linear regression model was obtained in the identification of mucinous type considering the 13 textural significant metrics extracted by the arterial phase (AUC of 0.93, accuracy of 94%, sensitivity of 77%, and specificity of 99%) and in the identification of tumor budding considering the 11 textural significant metrics extracted by the arterial phase (AUC of 0.92, accuracy of 93%, sensitivity of 94%, and specificity of 90%).

The coefficients of these linear models are reported in the Table 7.

#### 3.2.2. Pattern Recognition Approaches Findings

**Figure 4:**
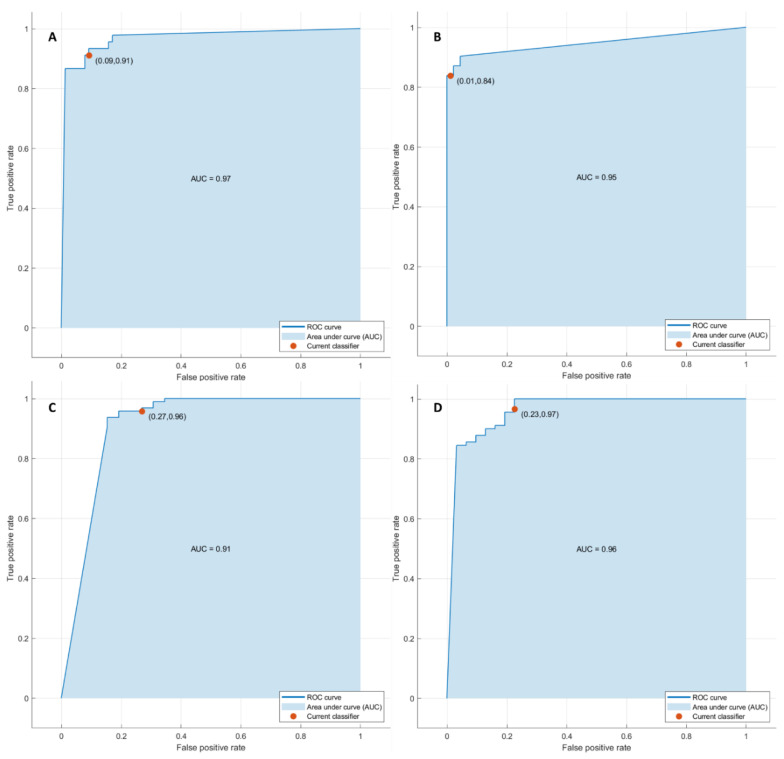
ROC curves of KNN with respect to the front of tumor growth (**A**), tumor budding (**B**), tumor mucinous type (**C**), and the recurrence presence (**D**) obtained considering significant features extracted by arterial phase.

Considering significant texture metrics tested with pattern recognition approaches, the best performance for each outcome (1. front of tumor growth: expansive versus infiltrative; 2. tumor budding: high-grade versus low-grade or absent; 3. mucinous type and 4. presence of recurrence) was reached by a KNN as classifier given the features extracted by the arterial phase (Figure 4). Instead, considering the features extracted by the portal phase, the best performance was reached by a KNN as classifier in the identification of the font of tumor growth, mucinous type, and for the detection of recurrences, and by a decision tree for the tumor budding identification (Table 5 and Table 6).

**Figure 5:**
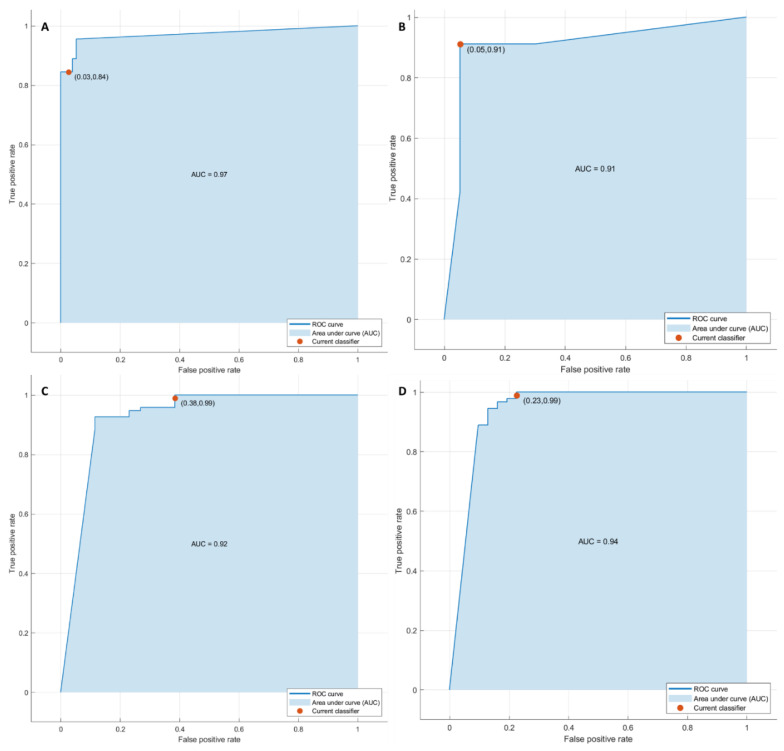
ROC curves of KNN with respect to the front of tumor growth (**A**), tumor budding (**B**), tumor mucinous type (**C**), and the recurrence presence (**D**) obtained considering significant features extracted by portal phase.

The accuracy was always greater than 88% (Table 5 and Table 6, Figure 5) in both the training and validation sets, and the best results were obtained in the identification of tumor budding, with the eleven textural significant features extracted by the arterial phase (AUC of 0.95, accuracy of 95%, sensitivity of 84%, and specificity of 99%).

The best performance in terms of accuracy obtained considering the extracted radiomics features on arterial phase was significantly superior at the best performance obtained in the portal phase (*p* value < 0.05 at McNemar test).

## 4. Discussion

The present study confirmed the possibility of radiomics to recognize as biomarkers several features that could influence the treatment choice in patients with liver metastases in order to obtain a more personalized approach. Our data were verified by an external validation dataset. We obtained a good performance considering the single textural significant metric in the identification of front of tumor growth (expansive versus infiltrative) and tumor budding (high-grade versus low-grade or absent), in the recognition of mucinous type, and in the detection of recurrences.

At univariate analysis, the best predictor to discriminate expansive versus infiltrative tumor growth front was wavelet_LHH_glrlm_ShortRunLowGrayLevelEmphasis by portal phase with accuracy of 82%. The best predictor to discriminate tumor budding was wavelet_LLH_firstorder_10Percentile by portal phase with accuracy of 92%. The best predictor to differentiate the mucinous type was the wavelet_LLL_glcm_ClusterTendency by portal phase with accuracy of 88%. The best predictor to identify the recurrence was wavelet_HLH_ngtdm_Complexity by arterial phase with accuracy of 90%.

The best linear regression model was obtained in the identification of mucinous type considering the 13 textural significant metrics extracted by the arterial phase (AUC of 0.93, accuracy of 94%, sensitivity of 77%, and specificity of 99%). The best results were obtained in the identification of tumor budding with the eleven textural significant features extracted by the arterial phase using a KNN (AUC of 0.95, accuracy of 95%, sensitivity of 84%, and specificity of 99%). Therefore, the best performance was reached considering the radiomics features extracted on arterial phase.

Several studies demonstrated the correlation between radiomics parameters and prognosis [31,32,33,34,35,36,37,38,39,40]. An association between homogeneity and worse overall survival (OS) was demonstrated by Andersen et al. [32]. According to Rahmim et al., radiomic parameters of heterogeneity obtained by FDG PET were predictors of lower OS [37]. Lubner et al. demonstrated that the degree of skewness was inversely correlated to KRAS, while the entropy was related with OS [34]. In addition to the survival advantages, the possibility to predict recurrence in the liver has been demonstrated [37,38,39,40]. According to our results, Ravanelli et al. related high CT uniformity and low OS and PFS in patients with CRC and liver metastasis [39].

Radiomics and radiogenomics are emerging tools with significant limits. The major weakness is the heterogeneity of software employed in different investigations due to the variety of imaging devices in different hospitals. This clearly hinders the interpretation of different data for multicenter studies. In addition, the segmentation may affect results [41].

Many previous studies have shown that the lower the degree of differentiation of the primary tumor (mainly manifested by more aggressive and malignant phenotype), the worse the prognosis; the number of metastases (≤ 4) and the diameter of metastases (≤5 cm) are the main prognostic factors affecting the prognosis of patients with liver metastases [42,43,44]. Ma et al. showed that the lower the degree of tumor differentiation, the greater the number of metastases, and the larger the diameter of the metastases, the heavier the tumor burden throughout the body, and the shorter the survival period. The distance between metastases and great vessels could affect the recurrence-free survival of patients [45]. However, the prognostic and predictive value of radiomics in colorectal cancer metastases to the liver have been well studied, demonstrating its higher utility in predicting clinical outcomes compared to other clinical data [2].

The present study had several limitations: (1) the small size of the population considered, although the analysis was performed on a homogeneous sample and on all individual lesions; furthermore the patients analyzed included a training set and an external validation set for a total of 151 liver lesions analyzed. The external dataset was used to validate the results obtained during the training phase; however, the results of this study were considered preliminary, and the future goal is to broaden the dataset to evaluate the generalization of the results; (2) the retrospective nature of the study; (3) a manual segmentation, that, although several studies support automatic segmentation to avoid inter-observer variability, in our opinion, the manual approach is more realistic. Moreover, we did not assess the impact of chemotherapy. However, we assessed the impact of the different phases of contrast study (arterial and portal), although we have not evaluated these results with respect to transitional and EOB-phase due to morphological sequences, such as T2-weigthed, T1-weigthed, or diffusion-weighted imaging. Data that we plan to evaluate in a future study.

## 5. Conclusions

Our results confirmed the capacity of radiomics to identify as biomarkers several prognostic features that could affect the treatment choice in patients with liver metastases in order to obtain a more personalized approach. We obtained a good performance considering the single textural significant metric in the identification of front of tumor growth (expansive versus infiltrative) and tumor budding (high-grade versus low-grade or absent), in the recognition of mucinous type and in the detection of recurrences.

## Figures and Tables

**Table 1:** Characteristics of the study population (81 patients).

Patient Description	Numbers (%)/Range
Gender	Men 53 (65.4%)
	Women 28 (34.6%)
Age	61 y; range: 35–82 y
**Primary cancer site**	
Colon	52 (64.2%)
Rectum	29 (35.8%)
**Prior Chemotherapy**	81 (100%)
**Hepatic metastases description**	
Patients with single nodule	52 (64.2%)
Patients with multiple nodules	29 (35.8%)/range: 2–13 metastases
Nodule size (mm)	mean size 36.4 mm; range 7–58 mm
**Front of tumor growth**	
Expansive	30 (37.0%)
Infiltrative	51 (63.0%)
**Tumor Budding**	
Absent	12 (14.8%)
Low grade	14 (17.3%)
High grade	55 (67.9%)
**Mucinous carcinoma**	25 (30.9%)
**Recurrence** **(new liver metastases)**	19 (23.5%)
**RAS mutation**	42 (51.9%)

**Table 2:** MR Sequence parameters.

Sequence	Orientation	TR/TE/FA (ms/ms/deg.)	AT (min)	Acquisition Matrix	ST/Gap (mm)	FS
Trufisp T2-W	Coronal	4.30/2.15/80	0.46	512 × 512	4/0	without
HASTE T2-W	Axial	1500/90/170	0.36	320 × 320	5/0	without and with (SPAIR)
HASTE T2w	Coronal	1500/92/170	0.38	320 × 320	5/0	without
In-Out phase T1-W	Axial	160/2.35/70	0.33	256 × 192	5/0	without
VIBE T1-W_FA10	Axial	4.80/1.76/10	0.18	320 × 260	3/0	with (SPAIR)
VIBE T1-W_FA30	Axial	4.80/1.76/30	0.18	320 × 260	3/0	with (SPAIR)

Note: W = weighted, TR = repetition time, TE = echo time, FA = flip angle, AT = acquisition time, SPAIR = spectral adiabatic inversion recovery, VIBE = volumetric interpolated breath hold examination, HASTE = half-Fourier-acquired single-shot turbo spin echo.

**Table 3:** (Sub)datasets, variable selection criteria and predictors combinations.

Dataset	Outcome Variable	Predictors	Accuracy Threshold on Univariate Analysis
Dataset 1	Front of tumor growth	Radiomic metrics on lesion by VIBE_FA10	≥0.75
Dataset 2	Tumor budding	Radiomic metrics on lesion by VIBE_FA10	≥0.80
Dataset 3	Mucinous Type	Radiomic metrics on lesion by VIBE_FA10	≥0.80
Dataset 4	Recurrence presence	Radiomic metrics on lesion by VIBE_FA10	≥0.80
Dataset 5	Front of tumor growth	Radiomic metrics on lesion by VIBE_FA30	≥0.80
Dataset 6	Tumor budding	Radiomic metrics on lesion by VIBE_FA30	≥0.85
Dataset 7	Mucinous Type	Radiomic metrics on lesion by VIBE_FA30	≥0.85
Dataset 8	Recurrence presence	Radiomic metrics on lesion by VIBE_FA30	≥0.85

**Table 4:** Findings by univariate analysis with ROC performance results.

Significant Textural Features Extracted	by Arterial Phase Respect to the Front of Tumor Growth	by Portal Phase Respect to the Front of Tumor Growth	by Arterial Phase Respect to the Tumor Budding	by Portal Phase Respect to the Tumor Budding	by Arterial Phase Respect to the Mucinous Type	by Portal Phase respect to the Mucinous Type	by Arterial Phase Respect to Recurrence	by Portal Phase Respect to Recurrence
	wavelet_LHH_glrlm_ShortRunLowGrayLevelEmphasis	wavelet_LHH_glrlm_ShortRunLowGrayLevelEmphasis	wavelet_LHH_firstorder_Minimum	wavelet_LLH_firstorder_10Percentile	wavelet_HLH_glszm_LargeAreaHighGrayLevelEmphasis	wavelet_LLL_glcm_ClusterTendency	wavelet_HLH_ngtdm_Complexity	wavelet_LLH_glcm_DifferenceEntropy
AUC	0.69	0.80	0.71	0.80	0.59	0.70	0.74	0.74
Sensitivity	0.95	0.84	0.98	0.96	0.35	0.38	0.71	0.71
Specificity	0.51	0.77	0.52	0.81	0.99	1.00	0.95	0.94
PPV	0.77	0.85	0.85	0.93	0.90	1.00	0.79	0.81
NPV	0.85	0.74	0.89	0.86	0.85	0.86	0.90	0.90
Accuracy	0.79	0.82	0.86	0.92	0.85	0.88	0.90	0.89
Cut-off	0.12	0.12	−41.76	−37.14	−0.02	408.22	3.34	1.54

**Table 5:** Linear regression and pattern recognition analysis with significant features from the arterial phase.

**Linear Regression of Significant Features Extracted by the Arterial Phase**	**AUC**	**Sensitivity**	**Specificity**	**PPV**	**NPV**	**Accuracy**	**Cut-off**
respect to the front of tumor growth	0.74	0.89	0.89	0.93	0.83	0.89	1.45
respect to the budding	0.92	0.94	0.90	0.97	0.85	0.93	1.38
respect to the mucinous type	0.93	0.77	0.99	0.95	0.94	0.94	0.37
respect to the recurrence	0.81	0.58	0.97	0.86	0.87	0.87	0.43
**Pattern Recognition Analysis with Significant Features**	**Dataset**	**AUC**	**Accuracy**	**Sensitivity**	**Specificity**	**Training** **Time [sec]**	**Model Type and Parameters**
The best classifier is a KNN considering significant features extracted on arterial phase respect each of outcome (front of tumor growth, budding, mucinous type, recurrence)	Training set	0.97	0.91	0.91	0.91	2.34	Weighted KNN; number of neighbors:10; distance metric: Euclidean; distance weight: squared inverse
	Validation set	0.96	0.89	0.85	0.91		
	Training set	0.95	0.95	0.84	0.99	4.27	
	Validation set	0.95	0.95	0.8	1		
	Training set	0.87	0.88	0.97	0.56	8.55	
	Validation set	0.91	0.91	0.96	0.73		
	Training set	0.96	0.92	0.97	0.77	10.38	
	Validation set	0.93	0.92	1	0.66		

**Table 6:** Results of linear regression and pattern recognition analysis with significant features from the portal phase.

**Linear Regression of Significant Features Extracted by The Portal Phase**	**AUC**	**Sensitivity**	**Specificity**	**PPV**	**NPV**	**Accuracy**	**Cut-off**
respect to the front of tumor growth	0.88	0.80	0.89	0.92	0.73	0.83	1.58
respect to the budding	0.82	0.93	0.67	0.83	0.86	0.83	1.50
respect to the mucinous type	0.88	0.77	0.96	0.83	0.94	0.92	0.36
respect to the recurrence	0.92	0.94	0.82	0.64	0.97	0.85	0.28
**Pattern recognition analysis results**	**Dataset**	**AUC**	**Accuracy**	**Sensitivity**	**Specificity**	**Training** **time [sec]**	**Model Type and parameters**
The best classifier is a KNN considering significant features extracted on portal phase respect to the front of tumor growth	Training set	0.96	0.90	0.91	0.89	13.4	Weighted KNN; number of neighbors:10; distance metric: Euclidean; distance weight: squared inverse
	Validation set	0.97	0.92	0.84	0.97	9.74	
The best classifier is a decision tree considering significant features extracted on portal phase respect to the budding	Training set	0.99	0.91	0.81	0.96		Maximum number of splits: 100 Split criterion: Gini’s diversity index Surrogate decision splits: Off Hyperparameter options disabled
	Validation set	0.97	0.93	0.84	0.97	3.4	
The best classifier is a KNN considering significant features extracted on portal phase respect to the to the mucinous type	Training set	0.89	0.93	0.8	1		Weighted KNN; number of neighbors:10; distance metric: Euclidean; distance weight: squared inverse
	Validation set	0.92	0.91	0.99	0.62	11.8	
	Training set	0.98	0.92	1	0.62		
The best classifier is a KNN considering significant features extracted on portal phase respect to the recurrence	Validation set	0.94	0.93	0.99	0.77	10.1	

**Table 7:** Linear regression model coefficients and intercept with respective *p* value.

Linear Regression of the Textural Features Extracted by the Arterial Phase with Respect to the Front of Tumor Growth	Coefficients	*p* Value	*p* Value
Intercept	−1.99	0.31	<0.000
wavelet_LHH_gldm_SmallDependenceLowGrayLevelEmphasis	33.14	0.19	
wavelet_LHH_firstorder_Minimum	0.01	0.02	
wavelet_LHH_glrlm_ShortRunLowGrayLevelEmphasis	−1.32	0.14	
wavelet_LHH_glrlm_ShortRunEmphasis	−3.32	0.14	
wavelet_LLH_glszm_SmallAreaLowGrayLevelEmphasis	2.11	0.03	
wavelet_HLH_glcm_MaximumProbability	19.52	0.00	
wavelet_HHH_gldm_SmallDependenceHighGrayLevelEmphasis	5.17	0.39	
wavelet_HHH_glrlm_ShortRunHighGrayLevelEmphasis	0.06	0.70	
**Linear regression of the textural features extracted by the arterial phase with respect to the tumor budding**	Coefficients	*p* value	*p* value
Intercept	−12.52	0.00	<0.000
original_glcm_Idn	31.70	0.00	
original_glcm_Idm	42.60	0.00	
original_glcm_Id	−56.44	0.00	
wavelet_LHH_firstorder_Minimum	0.02	0.00	
wavelet_LHH_firstorder_10Percentile	−0.06	0.40	
wavelet_LLH_glcm_MaximumProbability	1.88	0.16	
wavelet_LLH_glcm_Imc1	8.92	0.01	
wavelet_LLH_firstorder_10Percentile	0.00	0.74	
wavelet_LLH_glrlm_GrayLevelNonUniformityNormalized	−4.57	0.05	
wavelet_LLH_glszm_SmallAreaLowGrayLevelEmphasis	1.67	0.11	
wavelet_HLH_firstorder_10Percentile	0.44	0.00	
**Linear regression of the textural features extracted by the arterial phase with respect to the mucinous type**	Coefficients	*p* value	*p* value
Intercept	−2.18	0.01	<0.000
original_glszm_ZoneVariance	0.00	0.14	
original_glszm_LargeAreaEmphasis	0.00	0.11	
original_glszm_LargeAreaLowGrayLevelEmphasis	0.00	0.01	
wavelet_HLL_glcm_InverseVariance	4.62	0.01	
wavelet_HLL_glrlm_RunLengthNonUniformity	0.00	0.01	
wavelet_LHH_glszm_LargeAreaEmphasis	0.00	0.08	
wavelet_LHH_glszm_ZonePercentage	0.00	0.01	
wavelet_LHH_glszm_LargeAreaLowGrayLevelEmphasis	17.35	0.00	
wavelet_LHH_glszm_HighGrayLevelZoneEmphasis	0.00	0.00	
wavelet_LLH_glcm_InverseVariance	0.00	0.95	
wavelet_HLH_glcm_Imc1	0.61	0.64	
wavelet_HLH_glszm_LargeAreaHighGrayLevelEmphasis	11.35	0.00	
wavelet_HHH_glszm_ZonePercentage	0.00	0.00	
**Linear regression of the textural features extracted by the arterial phase with respect to the recurrence presence**	Coefficients	*p* value	*p* value
Intercept	0.44	0.11	0.030
wavelet_LHL_glcm_JointAverage	0.00	-	
wavelet_LHL_glcm_SumAverage	−0.20	0.08	
wavelet_LHL_glcm_MCC	0.26	0.65	
wavelet_LHL_glszm_SmallAreaHighGrayLevelEmphasis	−0.03	0.42	
wavelet_LHL_glszm_HighGrayLevelZoneEmphasis	0.07	0.04	
wavelet_LHL_ngtdm_Complexity	−0.02	0.48	
wavelet_LLH_firstorder_InterquartileRange	0.11	0.20	
wavelet_LLH_firstorder_RobustMeanAbsoluteDeviation	−0.25	0.22	
wavelet_LLH_ngtdm_Contrast	8.37	0.04	
wavelet_HLH_ngtdm_Complexity	0.03	0.07	
**Linear regression of the textural features extracted by the portal phase with respect to the front of tumor growth**	Coefficients	*p* value	*p* value
Intercept	−5.36	0.09	<0.000
wavelet_LHH_gldm_SmallDependenceLowGrayLevelEmphasis	−11.71	0.34	
wavelet_LHH_glrlm_ShortRunLowGrayLevelEmphasis	1.47	0.01	
wavelet_LHH_glszm_GrayLevelNonUniformityNormalized	0.14	0.78	
wavelet_LLH_firstorder_10Percentile	0.00	0.57	
wavelet_HLH_glcm_JointEnergy	23.11	0.06	
wavelet_HLH_glcm_MCC	1.22	0.11	
wavelet_HHH_glcm_MCC	16.45	0.00	
wavelet_HHH_glcm_Imc2	−9.75	0.04	
wavelet_LLL_firstorder_Uniformity	−0.50	0.47	
**Linear regression of the textural features extracted by the portal phase with respect to the tumor budding**	Coefficients	*p* value	*p* value
Intercept	29.69	0.06	<0.000
original_glrlm_GrayLevelNonUniformityNormalized	2.16	0.52	
original_glszm_ZoneVariance	0.00	0.02	
original_glszm_SmallAreaLowGrayLevelEmphasis	1.38	0.39	
wavelet_LHH_firstorder_10Percentile	0.18	0.00	
wavelet_LHH_ngtdm_Busyness	0.00	0.44	
wavelet_LLH_firstorder_10Percentile	0.02	0.00	
wavelet_LLH_glszm_LargeAreaLowGrayLevelEmphasis	0.00	0.23	
wavelet_LLH_glszm_SmallAreaLowGrayLevelEmphasis	5.37	0.06	
wavelet_HHH_glcm_JointEnergy	−111.34	0.09	
wavelet_HHH_glcm_MCC	16.23	0.00	
wavelet_LLL_glrlm_GrayLevelNonUniformityNormalized	−8.05	0.15	
wavelet_LLL_glszm_ZoneVariance	0.00	0.33	
wavelet_LLL_glszm_LargeAreaEmphasis	0.00	0.38	
**Linear regression of the textural features extracted by the portal phase with respect to the mucinous type**	Coefficients	*p* value	*p* value
Intercept	−0.10	0.51	<0.000
original_gldm_GrayLevelVariance	−2.92	0.05	
original_glcm_SumSquares	2.40	0.32	
original_glcm_ClusterProminence	0.00	0.16	
original_glcm_ClusterTendency	0.18	0.82	
original_firstorder_Variance	0.00	0.81	
original_glrlm_GrayLevelVariance	−0.62	0.00	
wavelet_LLL_gldm_GrayLevelVariance	1.73	0.03	
wavelet_LLL_glcm_SumSquares	−0.30	0.35	
wavelet_LLL_glcm_ClusterProminence	0.00	0.10	
wavelet_LLL_glcm_ClusterTendency	−0.02	0.87	
wavelet_LLL_firstorder_Variance	0.00	0.10	
wavelet_LLL_glszm_GrayLevelVariance	0.02	0.00	
**Linear regression of the textural features extracted by the portal phase h respect to the recurrence presence**	Coefficients	*p* value	*p* value
Intercept	−0.23	0.81	<0.000
wavelet_LLH_gldm_GrayLevelVariance	6.15	0.00	
wavelet_LLH_glcm_JointEntropy	−0.25	0.48	
wavelet_LLH_glcm_Contrast	−2.96	0.01	
wavelet_LLH_glcm_DifferenceEntropy	−4.97	0.05	
wavelet_LLH_glcm_DifferenceVariance	4.99	0.03	
wavelet_LLH_glcm_DifferenceAverage	9.93	0.00	
wavelet_LLH_firstorder_MeanAbsoluteDeviation	0.09	0.14	
wavelet_LLH_firstorder_RootMeanSquared	0.05	0.14	
wavelet_LLH_firstorder_Variance	−0.01	0.00	
wavelet_LLH_firstorder_Mean	0.04	0.00	
wavelet_LLH_glrlm_GrayLevelVariance	−1.06	0.34	

## Data Availability

Data and material are available at https://zenodo.org/deposit/6198703.
